# Knowledge, attitude, and practices of the community toward dengue fever in Shabwah Governorate, Yemen: a descriptive study

**DOI:** 10.1186/s42506-022-00121-5

**Published:** 2022-12-05

**Authors:** Mohammed Ali Saghir, Waled A. M. Ahmed, Mustafa Mohammed Abdullah Dhaiban, Murwan Eissa Osman, Naif Izzatullah Abduljabbar

**Affiliations:** 1 Community Medicine Department, Faculty of Medicine, International University of Africa, Khartoum, Sudan; 2 Nursing Department, Faculty of Applied Medical Sciences, Albaha University, Al Bahah, Saudi Arabia; 3 Malaria Eradication Program, Shabwah Health Office, Shabwah, Yemen; 4 Community Health Nursing Department, Faculty of Nursing, University of Khartoum, Khartoum, Sudan

**Keywords:** Knowledge, Attitude, Practice, Dengue fever, Yemen

## Abstract

**Background:**

In Yemen, dengue fever (DF) is a widespread, locally endemic infectious disease, with high morbidity and mortality which mainly affects impoverished regions. Thus, this study aims to evaluate the knowledge, attitudes, and behaviors of the Shabwah community in Yemen regarding DF characteristics and prevention.

**Methods:**

The present study is a cross-sectional community-based study conducted in Shabwah Governorate, Yemen, between June 2021 and November 2021. Simple random sampling was used to select the sample (*n* = 370), and a validated closed-ended questionnaire was used to collect data.

**Results:**

In total, 370 individuals participated in this study; most respondents were female (*N* = 247, 66.8%), and more than half (*N* = 214, 57.8%) were younger than age 30. More than 50% of the population had completed a primary or secondary school, while approximately 33.03% of them were illiterate. Furthermore, more than half (53.5%) of the population had good knowledge of DF, while most of those educated at a university level (64.1%) had good attitude toward DF. Most of the population (68.4%) had good practice toward DF. Moreover, participants with a university level education, followed by those who completed secondary school, had significantly higher knowledge and practice scores than primary school and illiteracy (*P* = 0.05).

**Conclusion:**

The results of the study indicate that the residents of Yemen’s Shabwah Governorate are well-informed about the symptoms and signs of DF, have a positive attitude toward the disease, and employ appropriate preventive measures. Health education via various media should be mandated to increase community awareness and dispel misconceptions about DF.

## Introduction

Dengue fever (DF) is the most prevalent disease transmitted by mosquitoes and is endemic to more than 100 countries. Approximately, 100 million cases and 25,000 deaths from DF occur annually [1–3].

Four serotypes of the dengue virus (DENV) can cause infection. The virus is spread by *Aedes* mosquitoes. *Aedes aegypti* mosquitoes are the main transmitters of DENVes [4]. The disease severity ranges from mild febrile illness to dengue hemorrhagic fever and shock syndrome [5].

Yemen has been adversely affected by the increasing frequency and geographic spread of epidemic dengue, and the number of reported cases has risen in tandem with the country’s social unrest and civil war [6, 7]. DF was first documented in the Shabwah Governorate in 1994, and since 2000, frequent outbreaks have emerged in Yemen. In Shabwah Governorate, outbreaks were recorded in 2001, 2002, 2005, 2018, 2019, and 2020 (unpublished reports), along with Aden and Taiz (2010, 2020), Hadramout/Mukalla (2005), and Al-Hudeidah Governorate, Yemen (1994, 2000, 2004, and 2005) [7, 8].

DF is a preventable viral infection; its successful prevention is contingent on the community’s knowledge, attitude, and practices (KAPs) regarding the disease. Despite the increasing incidence of DF in the Shabwah Governorate, no published study has documented these outbreaks and discussed the risk factors and the community’s knowledge, attitudes, and practices. This is the first study of KAP among the community in the Shabwah Governorate, which may be useful for understanding the gap and improving the disease’s control and prevention.

## Methods

### Study design and setting

The current study was a community-based cross-sectional study conducted between June 15 and November 30, 2021, in Ataq, the capital of the Shabwah Governorate. Shabwa is the third-largest governorate in Yemen by land area, located 474 km east of the capital Sana’a. Its geography consists of rugged mountains, plateaus, and valleys in the northwestern and central portions of the governorate, bordered by the Ramlat al-Sabatayn desert in the northeast and the coastal desert along the Arabian Sea. It has one of the lowest population densities in Yemen, but security improvements during the current war have attracted many people to settle primarily in the city of Ataq.

### Sampling technique and sample size

The individuals studied in the city were recruited using a simple random sampling method. The authors created a computer program to select the necessary sample size. The selection was made at random using the address entered into the computer software. According to the most recent census, there are 5000 houses in Ataq city. Therefore, the sample size was determined by Slovene’s formula:

$$n=\frac{N}{\left(1+N{e}^2\right)}$$

$$n=\frac{5000}{\left(1+5000\ast {0.05}^2\right)}=370\ \textrm{houses}$$

### Data collection instrument and method

A modified closed-ended interview questionnaire was used to collect data from the chosen residences. It consists of four sections: personal characteristics, knowledge, attitude, and practice of DF. The questionnaire includes 20 questions about the disease’s symptoms, transmission, risk factors, preventive measures, 6 statements about attitude, and 9 questions about practice. The authors requested a single respondent from each residence.

### Validation and pilot study

A preliminary test was conducted to evaluate the validity and dependability of the data collection instrument. Initially, the questionnaire was revised and corrected by three epidemiology and research specialists. The questionnaire was then distributed to 15 participants to determine the internal consistency reliability using Cronbach’s alpha. Cronbach’s alpha was greater than 0.70, indicating that the internal consistency reliability of the questionnaire was adequate.

### Statistical analysis

The collected data were analyzed by computer software using SPSS, version 26 (IBM corp. Armonk, NY, USA). Initially, all information collected via the questionnaire was initially coded into variables. The Kolmogorov-Smirnov test was used to ensure data normality. Subsequently, using descriptive and inferential statistics, the investigation was conducted. Spearman correlation tests were used to determine any correlation between the community’s knowledge, attitude, and practice, as well as to identify potential knowledge and practice determinants. It was determined that a relationship was significant when the *p*-value was less than 0.05.

### Score grading

A common grading method was used for each variable in this KAP questionnaire, as follows: knowledge was evaluated by asking 20 questions, the sum of which was calculated as the participant’s overall knowledge score (correct = 1, incorrect = 0), the overall score was between 0 and 20, and the cutoff point for good or bad knowledge was 50% (responses from 0 to 10 were considered as bad knowledge, and those scored more than 10 were considered as good knowledge). In order to evaluate the attitude of the community members toward the disease, six questions were posed, a 4-point Likert scale was used, and the scoring system was (strongly agree = 4, agree = 3, neutral = 2, and disagree = 1), the overall score was between 6 to 24, and the cutoff point for good or bad attitude was 50% (responses 12 or less were considered as bad attitude, and those scored more than 12 were considered as good attitude). Nine questions were posed concerning the practice of community members, with the same scoring system as for knowledge (correct = 1, incorrect = 0), the overall score was between 0 to 9, and the cutoff point for good or bad practice was 50% (responses from 0 to 4 were considered as bad practice, and those scored more than 4 were considered as good practice).

## Results

In total, 370 individuals participated in this study, most of the respondents were women (*N* = 247, 66.8). More than half (*N* = 214, 57.8%) of the study population was younger than 30 years old, 27.3% were between 30 and 40 years old, and 2.4% of respondents were older than 60 years old. More than 50% of the population had completed elementary or secondary school, while approximately 33% were illiterate. About half of the families were composed of less than 6 members (*N* = 174, 47.0%) (Table 1).

**Table 1:** Sociodemographic characteristics of study participants, Shabwah, Yemen (*n* = 370)

Variable	No. (%)
**Age**	
Less than 30	214 (57.8)
30–40	101 (27.3)
> 40–50	38 (10.3)
> 50–60	8 (2.2)
**> 60**	9 (2.4)
**Sex**	
Male	123 (33.2)
Female	247 (66.8)
**Educational level**	
Illiterate	112 (30.3)
Primary school	128 (34.6)
Secondary school	91 (24.6)
University	39 (10.5)
**Family members**	
< 6	174 (47.0)
7–10	129 (34.9)
> 10	67 (18.1)

Table 2 illustrates that the sample population’s mean knowledge score was 15.04 ± 2.87, with 53.5% of the population having good knowledge scores and 46.5% having poor knowledge scores. Those having good attitude toward DF (64.1%) were much more than those having bad attitude (35.9%). Higher percentage also have good practices toward DF prevention (68.4) compared to those who have bad practices (31.4%).

**Table 2:** Overall knowledge, attitude, and practices regarding DF in Shabwah, Yemen (*n* = 370)

Variables	Frequency	Percent
**Knowledge score**		
**Bad**	172	46.5
**Good**	198	53.5
**Attitude score**		
**Bad**	133	35.9
**Good**	237	64.1
**Practice score**		
**Bad**	117	31.6
**Good**	253	68.4

Table 3 shows knowledge of dengue symptoms and signs. The vast majority of participants (98.6%) mentioned fever, followed by headache (93.2%), joint pain (86.0%), muscle pain (78.4%), retro-orbital pain (74.5%), skin rash (45.5%), and bleeding (44.7%). More than half of the participants (57.9%) were aware that not all mosquitoes can transmit DF; three-quarters (75%) was aware that *Aedes* mosquitoes (black mosquitoes) transmit DF. Unfortunately, 50% of respondents believed that flies transmit the DENV. Regarding their beliefs, 43.2% cited contact with infected patients, and 46.5% cited food and water as modes of transmission of DF, whereas only 56% was aware that dengue mosquitoes are most likely to feed/bite during the day. Notably, most respondents (94.0% and 91.6%, respectively) were aware that stagnant water and keeping water containers open contribute to the spread of mosquitoes.

**Table 3:** Knowledge about DF in Shabwah, Yemen (*n* = 370)

Knowledge items				Correct response
**Dengue fever signs and symptoms**				
Fever				360 (98.6)
Headache				340 (93.2)
Joint pain				314 (86.0)
Muscle pain				286 (78.4)
Retro-orbital pain				272 (74.5)
Skin rash				166 (45.5)
Bleeding				163 (44.7)
**Transmission**				
Can all mosquitoes transmit DF?				213 (57.9)
Do the *Aedes* mosquitoes (black mosquitoes) transmit DF?				276 (75.0)
Flies do not transmit dengue				183 (49.7)
Contact with infected patients does not transmit DF				209 (56.8)
Is DF transmitted through food and water?				197 (53.5)
When are the dengue mosquitoes most likely to feed/bite?				206 (56.0)
**Factors mentioned to increase mosquito spread**				
Stagnant water				345 (94.0)
Keeping water containers opened				336 (91.6)
**Factors mentioned to reduce mosquito spread**				
Windows screens and bed net reduce mosquitoes				344 (93.7)
Insecticides sprays reduce mosquitoes and prevent DF				325 (88.6)
Tightly covering water containers reduces mosquitoes				352 (95.9)
Mosquito repellents prevent mosquito bites				337 (91.8)
Removal of standing water can prevent mosquito breeding				341 (92.9)
**Total**				
Minimum	Maximum	Median	Mean	SD
1	20	16	15.04	2.87

The percentage of the population who knew the correct answer for the questions on awareness of dengue prevention was as follows: tightly covering water containers reduces mosquitoes (95.9%), window screens and bed nets reduce mosquitoes (93.7%), removal of standing water can prevent mosquito breeding (92.9%), mosquito repellents prevent mosquito bites (91.8%), and insecticides sprays reduce mosquitoes and prevent DF (88.6%).

Table 4 shows that the mean attitude score of the sample population was 20.21 ± 2.7 out of 24 indicating that the participants had good attitude.

**Table 4:** Attitudes toward DF in Shabwah, Yemen (*n* = 370)

Attitude items		Strongly agree No. (%)	Agree No. (%)	Not sure No. (%)	Disagree No. (%)
Dengue fever is a serious disease		276 (74.6)	64 (17.3)	21 (5.7)	9 (2.4)
Dengue is a transmissible disease		124 (33.5)	115 (31.1)	50 (13.5)	81 (21.9)
I am at risk of dengue fever		165 (44.6)	107 (28.9)	68 (18.4)	30 (8.1)
Dengue fever can be prevented		44.6 (53.5)	28.9 (32.2)	18.4 (11.1)	8.1 (3.2)
Do you think that stagnant water around the houses in discarded tires, broken pots, and bottles is breeding places of *Aedes* mosquitoes?		244 (65.9)	111 (30)	13 (3.5)	2 (0.5)
Do you think communities should actively participate in controlling the vectors of DENV?		282 (76.2%)	78 (21.1%)	9 (2.4%)	1 (0.3%)
Total	Minimum	Maximum	Median	Mean^a^	SD
	9	24	21	20.21	2.66

^a^The range of the attitude score was from 9 to 24. The mean score was 20.2 and was calculated as follows: strongly agree 4, agree 3, neutral 2, and disagree 1; the cutoff point for good or bad attitude was 50%

In Table 5, the mean practice score of the surveyed population was 7.84 ± 1.5 out of a total score of 9 indicating that the surveyed population had good practices.

**Table 5:** Practices toward DF in Shabwah, Yemen (*n* = 370)

Practice items				Correct response No. (%)	
Use insecticide sprays to reduce mosquitoes				326 (88.1)	
Having mosquito nets				334 (90.3)	
Sleeping under mosquito nets				330 (89.2)	
Using fans for repelling mosquitoes				347 (93.8)	
Use screen windows to reduce mosquitoes				345 (93.2)	
Disposing water-holding containers such as tires, parts of automobiles, plastic bottles, and crack pots				342 (92.4)	
Using creams for repelling mosquitoes				240 (64.9)	
Covering body with clothes				285 (77.0)	
Cover water containers at home				350 (94.6)	
Total	Minimum	Maximum	Median	Mean	SD
	1	9	8	7.84	1.5

**Fig. 1:**
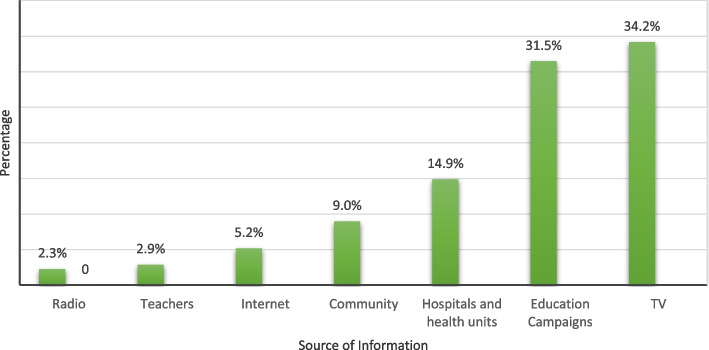
Sources of information regarding DF in Shabwah, Yemen (*n* = 370)

Figure 1 showed that television was the primary source of information about DF (34.2%), followed by education campaigns (31.5%), hospitals and health units (14.9%), the community (9%), the Internet (5.2%), teachers (2.9), and radio (2.3%).

Table 6 shows the significant correlations between knowledge and attitudes (*p*-value of 0.051), between knowledge and practices (*p*-value of 0.014), and attitudes and practices (*p*-value of 0.018).

**Table 6:** Spearman correlations between knowledge, attitudes, and practices (*n* = 370)

Variable(s)	***r***	***p***-value
Knowledge, attitudes	0.101	.051*
Knowledge, practices	0.127	.014*
Attitudes, practices	0.122	.018*

^*^Correlation is significant at the 0.05 level (2-tailed)

Table 7 shows that males have significantly higher knowledge score than females (*P* = 0.037), and that university level, followed by secondary school, had higher knowledge score than primary school and illiteracy (*P* = 0.01). Differences in practices were also statistically significant (*P* = 0.05) such that the university level, followed by secondary school, had higher practice scores than primary school and illiteracy, respectively.

**Table 7:** Mean rank of scores for knowledge, attitude, and practice in relation to personal characteristics (*n* = 371)

Variables	K. score 15.04 ± 2.86 Mean rank	***p***-value	A. score 20.21 ± 2.27 Mean rank	***p***-value	P. score 7.84 ±1.5 Mean rank	***p***-value
**Sex**^a^						
Male	201.76	.037*	193.17	0.327	186.73	0.867
Female	177.40		181.68		184.89	
Age^b^						
Less than 30	195.92	0.160	181.96	0.953	177.80	0.524
30–40	179.23		192.08		194.55	
41–50	157.36		185.84		200.17	
51–60	158.00		185.75		203.94	
> 60	151.44		194.06		188.72	
Education^b^						
Illiterate	169.53	.016*	175.00	0.148	171.28	.050*
Primary school	175.39		182.89		182.46	
Secondary school	206.99		187.24		192.43	
University	214.40		220.13		220.14	
Family members^b^						
< 6	162.78	0.175	155.10	0.978	159.43	0.882
7–10	160.17		157.25		155.53	
> 10	138.57		157.48		153.28	

*a* Mann–Whitney *U*-test, *b* Kruskal–Wallis test. *Significant at level 0.05, *K*, knowledge; *A*, attitude; *P* practice

## Discussion

Although DF is prevalent in Ataq, we discovered that only 53.5% of people have a good level of knowledge about DF, which is slightly less than what was reported in Westmoreland, Jamaica, in 2010 [9] and in a tertiary hospital in Sri Lanka in 2019 [10]. Our findings surpass those of previous studies conducted in Pakistan in 2010 [11], Malaysia in 2020 [12], and Indonesia in 2015 [13].

Although most respondents indicated that fever, headache, and joint pain are obvious symptoms of DF, and only two-thirds of respondents reported that muscle pain and retro-orbital pain are symptoms of DF, most respondents were unable to identify skin rash and bleeding as symptoms of DF. In comparable studies conducted in Taiz (Yemen) [6], Jamaica [9], Malaysia [7], and Cambodia [14], fever was also reported as the primary symptom of DF. This could be explained by educational messages in the mass media citing fever as dengue’s primary symptom [15] or by the participants’ personal experience with the disease or witnessing a close friend or relative’s case. Thus, raising awareness of these signs and symptoms could aid in distinguishing DF from other febrile infectious diseases, particularly in developing nations where DF is endemic.

More than half of the participants (57.9%) were aware that not all mosquitoes can transmit DF, three-fourths (75%) were aware that *Aedes* mosquitoes (black mosquitoes) can transmit the disease, and the majority (56%) were aware that dengue mosquitoes are most likely to feed/bite during the day. These results are lower than those of other studies conducted in Taiz, Yemen, which found that 82.2% of respondents believed that *Aedes* mosquitoes transmit DF, and that approximately two-thirds of respondents knew that these mosquitoes transmit DF primarily during the day [6]. In addition, in rural Cambodia, 96.7% of individuals were able to identify mosquitoes as the dengue vector, and 74% of participants believed that the dengue vector bites during the day [14]. Moreover, our findings demonstrated that respondents had greater knowledge than rural communities in Hodeidah, where approximately one-third of respondents perceived the daytime transmission of DF [16]. The high illiteracy rate (30.3%) of the Shabwah community in this study may be one of the reasons for the community’s lack of knowledge regarding the mosquito species that can transmit DF.

The study revealed that members of the Shabwah community had misconceptions regarding the transmission of DF, as they reported that the disease is transmitted by flies (50.3%), direct contact with an infected person (43.2%), drinking contaminated water, or eating contaminated food (46.5%). These misconceptions are higher than in previous studies conducted in Taiz; 80.7%, 85.1%, and 68% of participants correctly believed that flies contact with infected individuals, eating contaminated food, and drinking contaminated water played no role in the transmission respectively. In addition, a previous study conducted in Hodeidah revealed that most participants believed that the disease could be transmitted from an infected person to a healthy person via direct contact. A study in Bangladesh reported that only 6% of respondents believed that DF is transmitted through human-to-human contact [6, 16, 17].

Most participants in the current study recognized that stagnant water and keeping water in uncovered containers play a significant role in the transmission of DF by mosquitoes. These findings are comparable to those of previous research conducted in Taiz [6], Southern Thailand [18], and highland and lowland communities in Central Nepal [19]. The strengthening of mass media messages and educational campaigns in recent years [20] may have contributed to improved identification of risk factors.

This study revealed that the Shabwah community comprehensively understood dengue preventive measures and diverse breeding habitats. Likewise, several studies conducted in Taiz and rural Cambodia have found knowledge levels comparable to those of this study population [6, 14].

Despite proper knowledge of DF prevention, there is a gap in the perception of the transmission, necessitating intensive education campaigns to correct misconceptions and change behavior to reflect good knowledge in the practical life of the community, which may be crucial for maintaining the health status of families and communities.

Most participants in our study (64.1%) had a positive outlook in their attitude toward DF with no statistically significant correlation to socioeconomic factors. Similar to previous research conducted in Yemen’s Taiz and Hodeidah governorates, our study revealed a positive attitude regarding the severity and transmissibility of DF, as well as its prevention and community participation [6, 16].

In addition to covering the body with clothing, using creams to repel mosquitoes, and using insecticide sprays to reduce mosquito populations, the respondents adopted additional preventive DF measures. Contrary to previous studies conducted in Yemen, which reported low levels of community practice [6, 16], our study reveals a high level of community practice. This is likely because the practices in the community are primarily influenced by local tradition, culture, education, and exposure to other governorates in recent history.

We found a significant correlation between the practice of preventing DENV transmission and education level. The practice scores of those with university education, followed by those with secondary education, were higher than those with primary education and those who were illiterate.

Spearman correlations reveal a weak relationship between the KAP domains of respondents in this study. Although there was a significant correlation, the positive linear relationship between the three domains was weak; the correlation coefficients for each domain were less than 0.20. The most popular source of information about DF was television (34.2%), followed by education campaigns (31.5%), and radio. Unprecedented research in Central Nepal revealed that radio was the primary source of [19] information. Several studies conducted in Taiz, KSA, and Indonesia have reported similar primary sources [5, 6, 15].

### Limitations of the study

Due to the current internal conflicts and war, it is extremely difficult to travel between Yemen’s governorates to collect data, so this study has some limitations, such as a lack of generalizability. Another limitation is the use of the interview method for data collection which may be the reason for high score of the local community KAP toward DF.

## Conclusions

Most people in Shabwah, Yemen, have a solid understanding of the signs and symptoms of DF, have a positive outlook on various aspects of the disease, and employ appropriate preventive measures against DF. Health education via various media should be mandated to increase community awareness and dispel misconceptions about DF.

## Data Availability

Available upon reasonable request

## Abbreviations

DF: Dengue fever
DENV: Dengue virus
KAP: Knowledge, attitude, and practice
SPSS: Statistical Package of Social Sciences
